# Case Report: Pediatric chylous ascites beyond congenital malformations—infectious causes and nutritional management with a literature review

**DOI:** 10.3389/fped.2026.1779054

**Published:** 2026-03-05

**Authors:** Teresa Capriati, Annalisa Carciofi, Chiara Grimaldi, Andrzej Krzysztofiak, Simona Gatti, Maria Elena Lionetti, Michela Caprarelli, Annalisa Morelli, Lucia Tulli, Antonella Diamanti

**Affiliations:** 1 Digestive Diseases and Nutritional Rehabilitation, Bambino Gesù Children's Hospital, IRCCS, Rome, Italy; 2 Department of Pediatrics, Polytechnic University of Marche, Ancona, Italy; 3 Surgical Oncology Unit, Bambino Gesù Children's Hospital, IRCCS, Rome, Italy; 4 Infectious Diseases Unit, Bambino Gesù Children's Hospital, IRCCS, Rome, Italy

**Keywords:** case report, chylous ascites (CA), cytomegalovirus (CMV), lymphatic disease, parenteral nutrition (PN)

## Abstract

Chylous ascites (CA) is a condition characterized by the accumulation of lymphatic fluid in the peritoneal cavity. Although congenital malformations are the most common cause in newborns, infectious agents represent a clinically significant, potentially reversible etiology that benefits from specific therapy. Various pathogens, including bacteria, viruses, fungi, and parasites, can alter the lymphatic system and lead to the leakage of chyle into the peritoneal cavity, resulting in nutritional, immunological, and metabolic deficiencies. We describe the case of a 5-month-old infant presenting with acute abdomen (vomiting, irritability, and abdominal distension) associated with elevated lipase levels. He underwent emergency laparotomy, which revealed chylous ascites in the absence of structural abnormalities. Initial empiric management, centered on the diagnosis of CA, included fasting and total parenteral nutrition (TPN), followed by a gradual dietary transition from a lipid-free milk formula to a formula enriched in medium-chain triglycerides (MCTs) and reduced in long-chain triglycerides, which was well tolerated. Cytomegalovirus (CMV) infection was identified as the underlying cause and confirmed by PCR on blood, urine, ascitic fluid, and gastric biopsies. The etiological diagnosis allowed for specific antiviral therapy, which, combined with nutritional support, led to complete resolution of the case. We also review published cases of infectious CA in children, analyzing the clinical presentation, diagnostic approaches, and therapeutic strategies. Particular attention is paid to nutritional management. Interventions including the use of TPN, fat-free formulas, or MCT-enriched formulas are also important in infectious etiologies for temporarily controlling the chyle loss mechanism while awaiting complete lymphatic restitution. This review emphasizes the importance of recognizing infectious etiologies in chylous ascites and emphasizes the critical role of personalized nutritional support in optimizing recovery.

## 1 Introduction

Chylous ascites (CA) is a condition characterized by the accumulation of lymphatic fluid (chyle) in the peritoneal cavity. Chyle is a milky fluid primarily composed of chylomicrons, which result from the lymphatic absorption of long-chain triglycerides ingested in the diet. It also contains proteins and a variety of white blood cells, including T lymphocytes (1–3). CA is a rare condition in the pediatric population and is likely underdiagnosed. The exact incidence in children is unknown, but it ranges from 1 in 20,000 to 1 in 187,000 hospital admissions (3). In a recent review of 351 cases of fetal ascites, CA accounted for 6% of fetal ascites and had a perinatal survival rate of 100%⁴ (4). If not recognized and treated promptly, CA can evolve into a chronic and potentially serious condition, leading to the loss of nutrients, electrolytes, and immunoglobulins, resulting in malnutrition, immunosuppression, and metabolic disorders (1–3). The reported mortality rate for CA can be as high as 40%–70% (1).

The causes of CA can be congenital, acquired, or idiopathic. In infants and children, congenital forms are more common and can be a complication of complex syndromes (3, 5–8). In older children and adolescents, acquired forms include iatrogenic lesions of the thoracic duct or mesenteric lymphatics, trauma, lymphatic malignancies such as lymphoma and neuroblastoma, and vascular anomalies including generalized lymphatic anomalies (GLA), central lymphatic conduction anomalies (CCLA), Gorham-Stout disease (GSD), and kaposiform lymphangiomatosis (KLA). It can also manifest as part of syndromes associated with lymphatic malformations, such as Down, Turner, Noonan, and Hennekam, lymphatic obstruction due to abdominal masses, and inflammatory or infectious processes. In a small percentage of cases, the cause remains unknown, and the condition is classified as idiopathic (3).

The pathogenesis of CA is complex and not fully understood (9–11). A key contribution comes from radiological techniques that allow dynamic study of lymphatic flow (12–15). Lymphatic drainage occurs through three major networks: the retroperitoneal (20%), mesenteric (40%), and hepatic (40%) networks. These converge in the cisterna chyli, the central hub below the diaphragm (12). Obstruction of flow pathways and failure to empty the cisterna chyli can lead to CA. One mechanism involves the exudation of lymphatic fluid from acquired or congenitally dilated retroperitoneal vessels (megalymphatics) into the abdominal cavity through a fistula. These megalymphatics can form secondary to obstructions of the thoracic duct, often due to trauma, causing direct leakage through a lymphoperitoneal fistula. A bowel malignancy can also lead to fibrosis of the primary intestinal lymph node, obstructing lymph flow from the intestine to the cisterna chyli and resulting in leakage from dilated subserosal lymphatics. Over time, increased pressure can lead to collagen deposition in the basement membrane of lymphatics, further compromising intestinal absorption (9–11). This mechanism can also be triggered by infectious or inflammatory conditions affecting the mesenteric lymph nodes. Additionally, portal hypertension or right heart failure secondary to surgery (16) or myocarditis (12–15) can cause CA.

**Figure 1:**
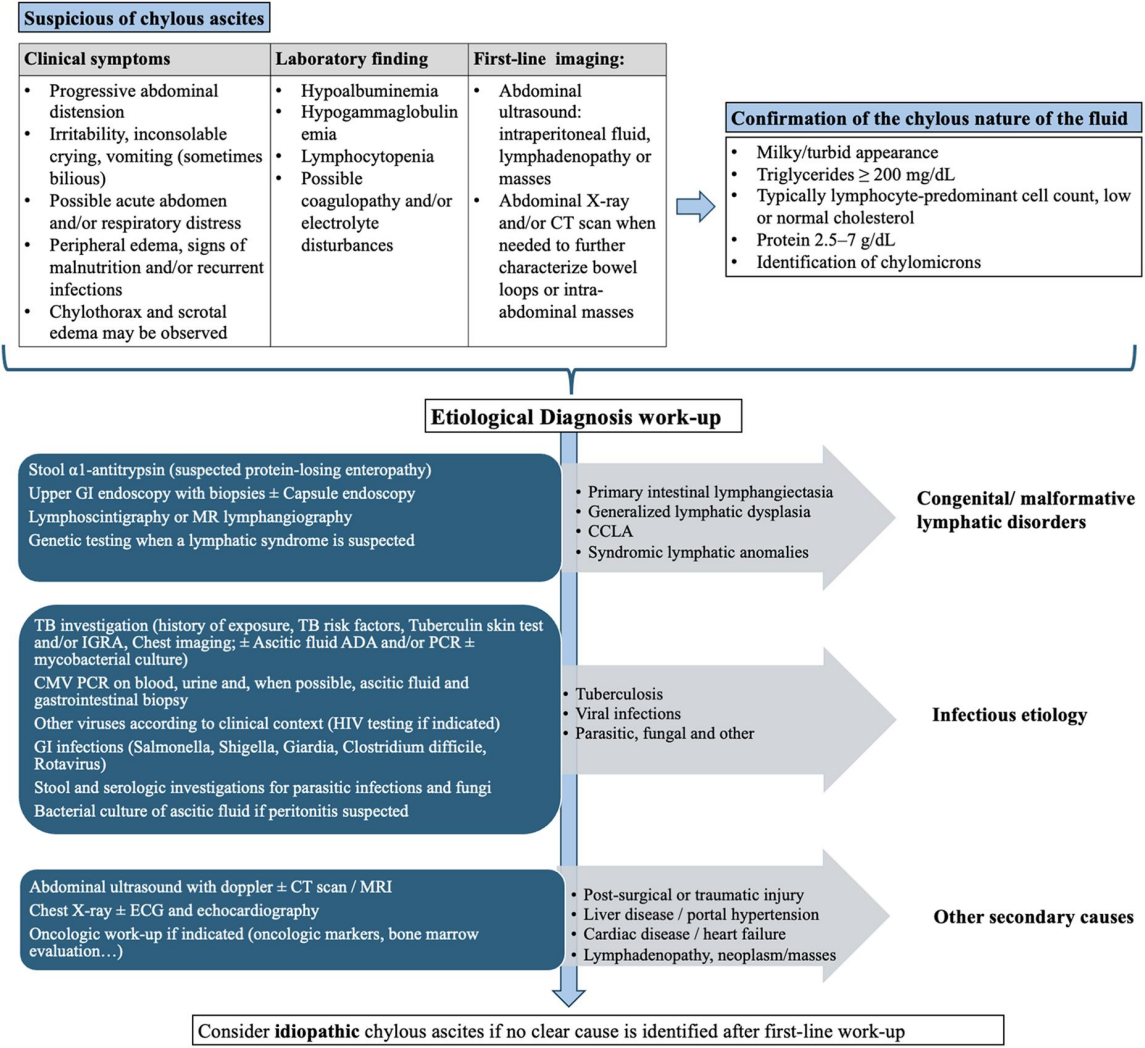
Diagnostic flowchart for suspected chylous ascites. CT, computed tomography; LDH, lactate dehydrogenase; GI, gastrointestinal; MR/MRI, magnetic resonance imaging; TB, tuberculosis; IGRA, interferon-gamma release assay; ADA, adenosine deaminase; PCR, polymerase chain reaction; CMV, cytomegalovirus; HIV, human immunodeficiency virus; ECG, electrocardiogram; CCLA, complex congenital lymphatic anomalies.

The diagnosis of CA is based on clinical history, laboratory findings, paracentesis, and ascitic fluid analysis. Clinical manifestations vary and correlate with fluid volume. Rapid accumulation can lead to an acute abdominal presentation similar to appendicitis (2, 3), but the most common symptom is progressive abdominal distension, often accompanied by weight gain and respiratory difficulty from the diaphragm being pushed upward (17). This may be associated with poor weight gain and a reduction in lean body mass due to loss of protein and fat. In some cases, recurrent infections due to immunoglobulin depletion and coagulation abnormalities occur. Chylothorax may coexist, and scrotal edema can be seen in male patients (18, 19). Laboratory findings typically show hypoalbuminemia, hypogammaglobulinemia, and lymphopenia. Coagulation abnormalities may be present due to the loss of factors and antithrombin. Analysis of ascitic fluid typically reveals a milky, cloudy fluid due to chylomicrons. Analysis usually shows normal cholesterol, elevated triglycerides (>200 mg/dL), high cellular count (>500 cells, predominantly lymphocytes), LDH >160 U/L, total protein between 2.5 and 7 g/dL, and glucose <100 mg/dL. Figure 1 shows the diagnostic work-up.

Advanced imaging plays a key role in assessing the site of chyle leakage. Ultrasound, a first-line technique, detects ascitic fluid but cannot identify the obstruction or site of leakage, which requires dynamic testing. Scintigraphic techniques, such as lymphoscintigraphy or lymphangiography with intranodal injection, allow visualization of lymphatic flow and identification of obstruction sites. However, the gold standard is dynamic contrast-enhanced magnetic resonance (MR) lymphangiography, which involves ultrasound-guided gadolinium injection into the inguinal lymph nodes. This technique provides excellent contrast distribution, allowing identification of both the leak site and complete lymphatic anatomy. It is a comprehensive, radiation-free procedure but requires specialized skills and anesthesia in most children (3, 12–15, 18). Esophagogastroduodenoscopy with biopsies may be useful to assess gastrointestinal involvement and to investigate possible infectious, inflammatory, infiltrative, or lymphatic/malformative conditions.

In the diagnostic workup, it is crucial to perform infectious disease investigations. Clinical suspicion may arise if symptoms compatible with infection precede the onset of CA. These tests are simple, minimally invasive, and can be performed immediately after clinical evaluation. Among acquired forms, infectious causes of CA are rare and more common in developing countries (19). Table 1 summarizes reported infectious pediatric CA cases in the literature (20–30). The main etiologies include peritoneal tuberculosis, followed by viral infections such as cytomegalovirus (CMV) and systemic parasitic diseases. The pathogenic mechanism is the obstruction or rupture of abdominal lymphatics.

**Table 1:** Infectious cases of CA in pediatric literature (etiology, therapeutic and nutritional management and outcome).

Author (year)	Age	Infectious agent	Etiologic therapy	Nutritional and medical therapy for CA	Outcome
Jhittay et al. (1986) (22)	5 years	Mycobacterium tuberculosis	Anti TB therapy (not reported)	Not reported	Resolution
Rabie et al. (2010) (26)	3 years	Mycobacterium tuberculosis (TB–IRIS in an HIV-positive child)	Rifampicin, isoniazid, pyrazinamide, ethambutol, ethionamide+HAART; corticosteroids;	Low-fat diet (later more stringent fat restriction); spironolactone Subcutaneous octreotide (200 mcg/dose 12 hly); no TPN;	Gradual resolution, full recovery
Azoumah et al. (2013) (23)	11 months	Mycobacterium tuberculosis	Isoniazid, rifampicin, pyrazinamide, ethambutol	Not reported	Complete resolution
Lee et al. (2013) (25)	19 days	Congenital Tuberculosis	Anti TB drugs (not reported)	Not reported	Clinical improvement
Kim et al. (2014) (21)	17 years	Mycobacterium tuberculosis	Isonia­zid, rifampin, pyrazinamide, ethambutol	High protein and low fat diet with MCT;	Clinical improvement
Mohammad et al. (2019) (20)	4 years	Mycobacterium tuberculosis	Isoniazid, rifampicin, ethambutol and pyrazinamide (2 months) → isoniazid and rifampicin (4 months)	MCT based diet	Full recovery, no recurrence
Birajdar et al. (2021) (24)	2.5 months (preterm)	Mycobacterium tuberculosis (perinatal TB)	Isoniazid, rifampicin, pyrazinamide and ethambutol	MCT-based formula for 12 weeks; Octreotide infusion (7 days).	Complete resolution
Berkowitz and Nesheim. (1993) (27)	2 years and 8 months	Mycobacterium avium complex in HIV-positive child	Rifampin, ethambutol, amikacin; dapsone, zidovudine; ciprofloxacin, clofazamine	Low LCT and high MCT diet; Spironolactone	Complete resolution
Santos et al. (2023) (28)	16 years	Paracoccidioides spp. (juvenile paracoccidioidomycosis)	Amphotericin B deoxycholate → liposomal amphotericin B (L-AmB) → itraconazole → L-AmB; sulfamethoxazole–trimethoprim; prednisone	Low-fat oral diet; octreotide (16 days)	Complete recovery
Leong et al. (1998) (29)	4 years	Ascaris lumbricoides	Mebendazole for 3 days	Total parenteral nutrition (TPN) 4 days, followed by low-fat, high-protein diet; Vitamin K supplementation;	Resolution; normal lymphography at 1 year
Greydanus et al. (1977) (30)	5 years	Cytomegalovirus (CMV)	No specific antiviral treatment	No specific treatment reported	Full recovery
Our case	5 months	Cytomegalovirus (CMV)	IV ganciclovir → oral valganciclovir (4 weeks)	Initial TPN → Low-fat formula → MCT-enriched formula;	Full recovery, no recurrence

Peritoneal tuberculosis is the most common infectious cause of CA in childhood. Obstruction of the collectors is due to granulomatous tuberculous lymphadenopathy. The first description involved a 2-year-old child (Morton 1964) (6, 10). Diagnosis is confirmed by detecting elevated adenosine deaminase activity in the ascitic fluid (20) or a positive GeneXpert test for Mycobacterium tuberculosis (24). Specific antituberculosis therapy combined with supportive and nutritional therapy is essential for recovery.

The viral etiology of CA is mostly associated with CMV (30). CMV infection is confirmed by an increase in antibody titers and viral isolation from urine. The mechanism is mesenteric lymphadenopathy causing obstruction of lymphatic flow. Conservative treatment without antiviral therapy has been reported, with spontaneous regression of abnormalities within a few weeks. Phen et al. (31) described a pediatric case of CA occurring after SARS-CoV-2 infection, secondary to heart failure. In this case, CA was due to hemodynamic consequences of significant systolic dysfunction with increased central venous pressure.

Parasitic and fungal infections are rare causes of CA in children, with cases linked to paracoccidioidomycosis (28) and ascariasis (29). In adults, CA has also been associated with filariasis (32). In parasitic and fungal infections, CA is often associated with chylothorax (28, 29), and lymphatic obstruction results from inflammatory lymphadenopathy. In these cases, in addition to therapeutic paracentesis, specific etiological treatments and corticosteroid therapy (prednisone) are essential to reduce lymph node obstruction.

The management of CA requires a multidisciplinary approach. In addition to its diagnostic role, paracentesis also serves a therapeutic purpose for large-volume CA. Nutritional therapy is the first-line conservative treatment for all forms of CA (primary, secondary, or idiopathic), as it reduces chyle production. For congenital forms with low chyle output, and certain infectious forms, this approach may resolve symptoms. The dietary strategy involves a low long-chain triglyceride (LCT) diet, replacing LCTs with medium-chain triglycerides (MCTs), which are absorbed directly into the portal circulation. Implementing this diet in children can be challenging due to issues with compliance. Additionally, careful monitoring of the child's nutritional and vitamin status is essential. In some cases, temporary parenteral nutrition may be required. Pharmacological management may involve periodic intravenous administration of albumin to compensate for protein losses. Somatostatin analogs, such as octreotide, are also used to reduce lymphatic flow and decrease portal pressure. In cases of diffuse or extensive lymphatic malformations, the use of immunosuppressive drugs like sirolimus (an mTOR inhibitor) or everolimus has shown promising results (1, 3). Additionally, propranolol and tranexamic acid may be useful in certain conditions, such as primary intestinal lymphangiectasia.

## 2 Case description

A 5-month-old male term born infant was transferred to our tertiary center for acute abdominal symptoms, including inconsolable crying, bilious vomiting, and abdominal distension. Family and perinatal histories were unremarkable. The infant was mixed-fed. Initial tests showed mild neutrophilic leukocytosis and elevated lipase (566 U/L) with normal pancreatic amylase, transaminases, and electrolytes. Abdominal radiographs showed dilated loops in mid abdomen; ultrasound revealed a large amount of ascites with echogenic strands and inflammatory changes in the adipose tissue. Given the clinical deterioration and suspecting intestinal volvulus, an emergency laparotomy was performed.

Abundant CA was identified, without evidence of volvulus, malrotation, or obstruction. Diffuse white peritoneal deposits were described and harvested for histological examination. Mesenteric fat histology revealed multifocal necrosis without neoplastic or vascular abnormalities. Two abdominal drains were placed. Peritoneal fluid analysis showed markedly elevated lipase (3,000 U/L), suggestive of pancreatic involvement, alongside increased LDH, triglycerides, and total protein.

The patient required intensive care, parenteral nutrition, antibiotics, and ventilatory support for approximately 48 h, leading to serum lipase normalization and progressive CA resolution. Postoperative day 1 CT scan is shown in Supplementary Material. PCR for cytomegalovirus (CMV) in ascitic fluid was positive. CMV testing in serum (100 copies/mL) and urine (132,230 copies/mL) on postoperative day (POD) 17 confirmed postnatal infection, as the birth urine test was negative.

Lymphoscintigraphy showed normal lymphatic drainage, excluding primary causes. Endoscopy revealed no mucosal abnormalities, and immunohistochemical staining for CMV was negative. However, CMV PCR of gastric tissue showed 2,390 copies per 100,000 cells, indicating active viral replication in gastric mucosa. Following specialist recommendations, intravenous ganciclovir was initiated for 8 days, then switched to oral valganciclovir (20 mg every 12 h) for 3 weeks.

**Figure 2:**
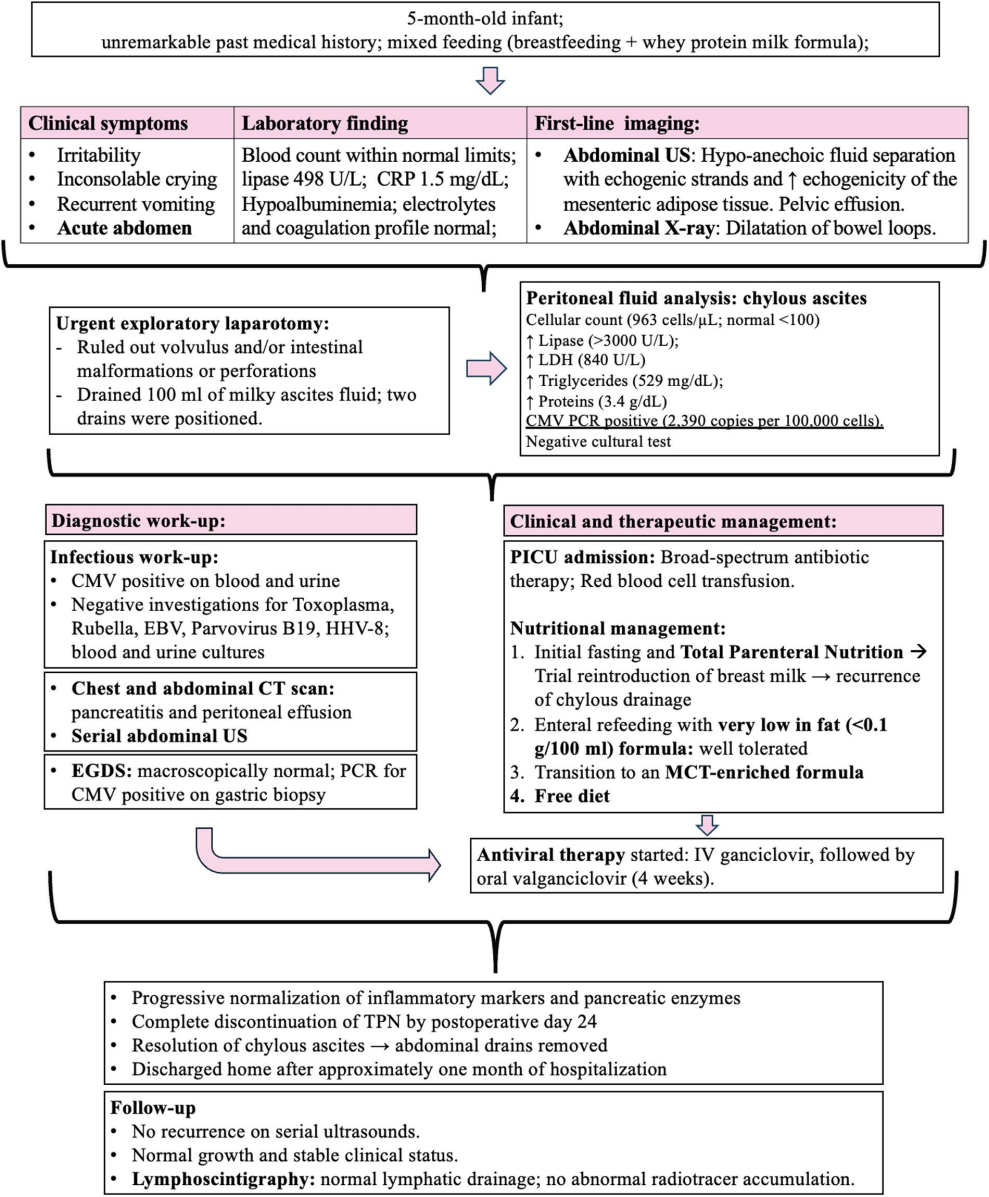
US, ultrasound; CRP, C-reactive protein; LDH, lactate dehydrogenase; CMV, cytomegalovirus; PCR, polymerase chain reaction; EBV, epstein–barr virus; HSV, herpes simplex virus; CT, computed tomography; EGDS, esophagogastroduodenoscopy; PICU, pediatric intensive care unit; TPN, total parenteral nutrition; MCT, medium-chain triglycerides.

Initially, the patient received total parenteral nutrition (TPN). Breast milk reintroduction on POD 6 triggered CA recurrence by POD 11. After resuming fasting, feeding restarted on POD 15 with low-fat formula, then switched to medium-chain triglycerides (MCT) formula on POD 21. TPN was discontinued by POD 24. Drainage remained non-chylous, and drains were removed on PODs 18 and 21. Complementary feeding began at 6 months alongside MCT formula (see patient's diagnostic and therapeutic flow chart in Figure 2).

**Figure 3:**
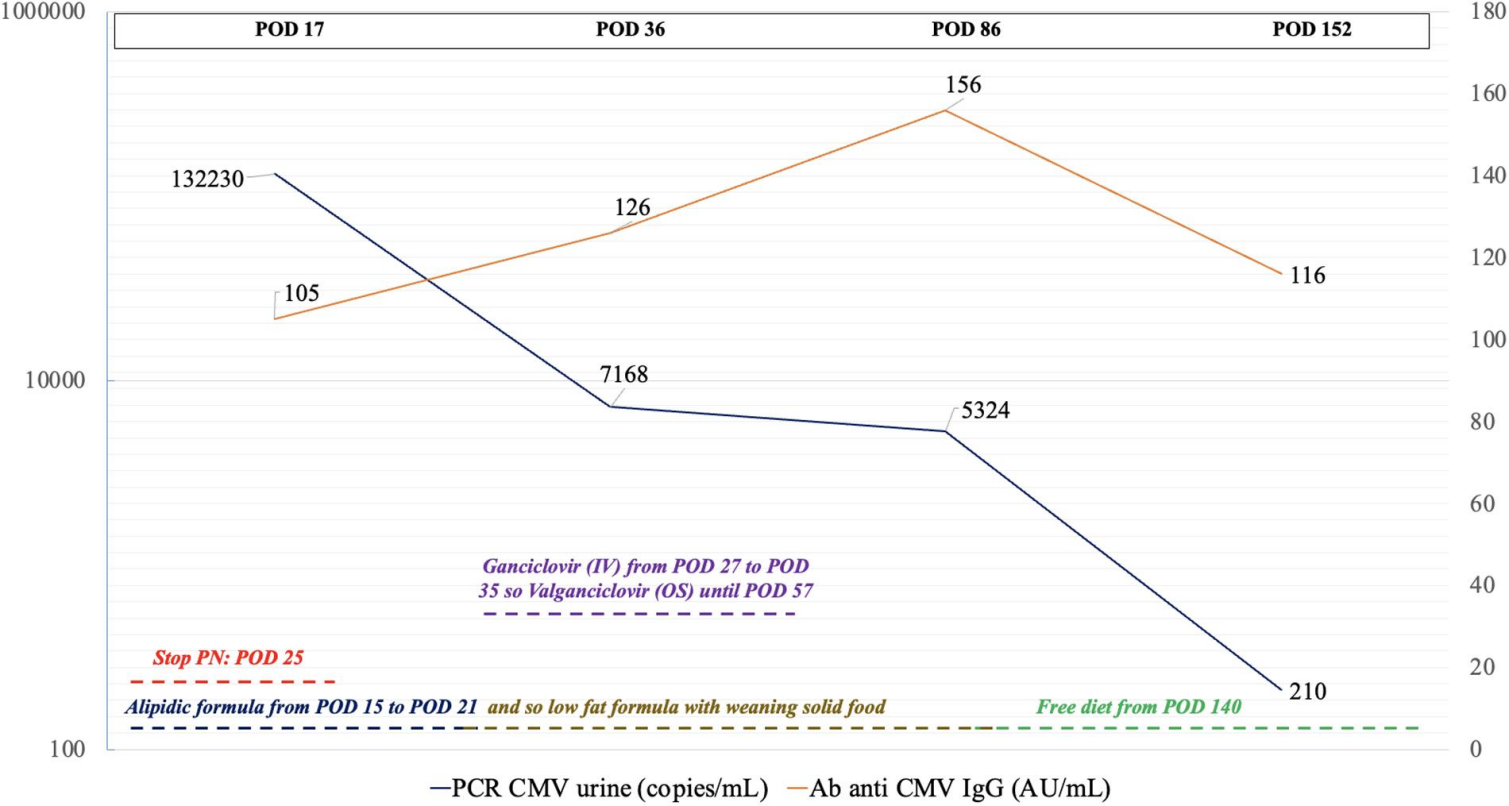
POD, postoperative day; PN, parenteral nutrition; IV, intravenous.

The patient maintained adequate growth during hospitalization: weight remained around the 15th percentile (7.0 kg at admission→ 7.43 kg at discharge), while length increased from 65 cm (15th percentile) to 69 cm (50th percentile), with weight-for-length stable at the 15th percentile (CDC charts). Hematological and pancreatic markers were normal. CMV DNA in urine decreased post-discharge with 210 copies/mL at 3 months post-surgery (see Figure 3). Saliva PCR became negative 40 days later. Initial elevation of fecal 1-antitrypsin normalized within a month. Follow-up ultrasounds showed no CA recurrence. At the final checkup, the child was free-feeding, up to date on vaccinations, and clinically stable with normal audiometric and ophthalmologic examinations.

## 3 Discussion

In children, CA is primarily linked to congenital or malformative lymphatic disorders, which account for 84% of cases (34). Infectious causes are rare and sporadically reported (1–3, 20–30). Tuberculosis is the most common infectious cause of CA and/or chylothorax (in adults up to 10% of atraumatic CA cases) (34). Viral (CMV) and parasitic (paracoccidioidomycosis, ascariasis) infections are less common but clearly documented.

The underlying mechanism for chyle leakage in tuberculosis is lymphatic obstruction due to granulomatous lymphadenopathy (20–27). In viral and parasitic infections, the damage may stem from lymphadenitis, lymphatic obstruction, or from severe systemic diseases with secondary involvement of the lymphatic system (28–30, 32).

We have reported a review of the literature on pediatric CA secondary to infectious processes (20–30) and in this context, we have described a case of acute presentation CA related to CMV infection with concomitant pancreatitis in an otherwise healthy infant and without primary structural lymphatic abnormalities. In all these cases, identifying the infectious cause is crucial, as it allows for targeted treatment that can resolve the chylous effusion.

The comprehensive review of cases underscores that systematic infectious investigations are crucial for diagnosing acute-onset CA, especially when “idiopathic” (35–37). Since many reports lack complete data, infectious causes must be ruled out in infants and children lacking surgical history, trauma, or congenital malformations (3, 6, 19). Alongside standard biochemical and infectious analyses of ascitic fluid, a structured infectious screening should include tests for tuberculosis, CMV, and parasitic infections, including mycobacterial cultures, viral PCR, and, where appropriate, microbiological and parasitological tests of blood, urine, and stool (20, 24, 29, 30).

Another crucial topic is that nutritional management is a critical therapeutic—not merely supportive—intervention in infection-related CA, as reducing lymphatic flow remains the primary symptomatic relief while awaiting resolution. However, the lack of randomized controlled trials and the diagnostic imaging complexity of the lymphatic system, often requiring specialized centers, mean that treatment protocols still rely on low-level evidence from case reports. In reviewed cases, dietary interventions varied in both approach and invasiveness—ranging from low-fat diets and restriction of long-chain triglycerides to the use of MCT-based formulas and, when necessary, temporary parenteral nutrition. Consistently essential for clinical improvement, these variable interventions underscore that individualized nutritional therapy—combined with targeted antimicrobials—is vital for resolution of chyle loss (20, 21, 24, 26, 28, 29), restoring metabolic balance, preventing protein loss, and supporting growth in infection-induced CA (1–3).

Our patient required initial total parenteral nutrition (TPN) to minimize lymphatic flow, as early reintroduction of breast milk triggered immediate chyle production. A phased enteral strategy—transitioning from a very low-fat formula to an LCT/MCT-enriched diet—enabled TPN discontinuation while maintaining growth. Following resolution and drain removal, the patient remained stable post-discharge. The absence of recurrence during dietary liberalization and the exclusion of congenital anomalies confirm a transient, infection-related etiology.

About CMV etiology, our case aligns with findings by Greydanus et al., where CMV-associated CA resolved via conservative management (30).

In our case, CMV involvement was confirmed through molecular analysis of blood, urine, ascitic fluid, and gastric tissue. Notably, while endoscopy appeared normal, PCR of gastric biopsies detected active viral replication (despite negative immunohistochemistry), confirming gastrointestinal involvement and a virus-induced lymphatic inflammatory disorder. This evidence, alongside the patient's age and hypogammaglobulinemia, led to a multidisciplinary decision to initiate ganciclovir/valganciclovir therapy, resulting in clinical and laboratory normalization.

Furthermore, in our case the infection coincided with acute pancreatitis, meeting International Study of Pancreatitis and Its Related Events (INSPPIRE) and North American Society for Pediatric Gastroenterology, Hepatology and Nutrition (NASPGHAN) criteria through elevated enzymes and characteristic imaging (38, 39).

Acute and chronic pancreatitis accounts for approximately 4% of atraumatic chylous ascites (CA) cases (34, 40–43). Although CA usually is a delayed complication occurring weeks post-episode (42, 44, 45), acute presentations have been documented, including cases with atypical biochemical profiles such as isolated lipase elevation or normal amylase (46–48). Pathogenetically, retroperitoneal inflammation may compress or disrupt lymphatic channels, leading to hypertension and lymph extravasation. Additionally, direct enzymatic damage to the lymphatic system (42) or secondary superior mesenteric-portal vein thrombosis—occurring in 7% of cases—can further exacerbate lymphatic obstruction (34, 49).

Another interesting topic is that, in addition to CA, our patient exhibited transient Protein-Losing Enteropathy (PLE), evidenced by an initial elevation of fecal 1-antitrypsin that normalized within a month. PLE involves an uncompensated loss of plasma proteins into the gut, caused by either direct mucosal damage (erosive or non-erosive) or impaired lymphatic drainage leading to epithelial backflow (50, 51). These pathways underpin a broad spectrum of PLE, ranging from inflammatory conditions like Crohn's disease and ulcerative colitis to cardiovascular disorders such as chronic congestive heart failure. Additionally, widespread abdominal malignancies, including lymphoma and multiple myeloma, can further disrupt these mechanisms, leading to significant protein loss.

In this context, CMV is thought to induce foveolar hyperplasia and increase epithelial permeability via abnormal tight junctions, facilitating protein leakage but also lymphadenitis and impaired lymphatic drainage. It is plausible that the same lymphatic disruptions—hypertension and failed drainage—that drive PLE can, in specific cases, lead to the extravasation of chyle into the peritoneal cavity. Whether a patient develops PLE, CA, or both likely depends on a combination of mechanical factors and the localized intensity of the viral-induced inflammatory response. CMV-associated PLE is often considered benign and self-limiting in immunocompetent children but recent literature (33) highlights that a significant subset—particularly infants around 2.7 months of age—can experience a severe clinical course.

## 4 Conclusions

In children, while CA is predominantly linked to congenital lymphatic malformations (34). The infectious etiologies—though rare—must be systematically considered. The main causes of pediatric CA, secondary infectious etiologies, are tuberculosis, CMV, and parasitic infections (20–30) and should always be actively investigated, especially in acute presentations without structural abnormalities (1–3).

The identification of CMV through highly sensitive molecular techniques (PCR), even in the absence of macroscopic endoscopic lesions or positive immunohistochemistry, highlights the critical role of viral screening in “idiopathic” presentations (35–37). Our findings suggest that CMV can trigger a complex clinical spectrum involving both Protein-Losing Enteropathy (PLE) and CA, likely driven by a combination of direct mucosal injury and transient lymphatic hypertension due to inflammatory lymphadenitis. Furthermore, the concomitant presence of acute pancreatitis underscores the systemic impact of CMV and its potential to exacerbate lymphatic obstruction through retroperitoneal inflammation (33, 50).

Effective management requires a multidisciplinary approach where individualized nutritional therapy—initially via TPN and subsequently through a graded MCT-enriched diet—acts as a primary therapeutic intervention to reduce lymphatic flow and restore metabolic balance (1–3). When combined with targeted antiviral treatment, this conservative strategy can lead to complete resolution without recurrence.

In conclusion, infectious screening should be integrated into the standard diagnostic workup for pediatric CA. Early detection of a transient infectious cause can prevent unnecessary invasive procedures, guide specific therapy, and ensure an excellent clinical prognosis in otherwise healthy infants.

## Data Availability

The raw data supporting the conclusions of this article will be made available by the authors, without undue reservation.
