# Supplementary figures and images for: Case Report: Pediatric chylous ascites beyond congenital malformations—infectious causes and nutritional management with a literature review
Source: Front Pediatr. 2026 Mar 5;14:1779054. doi: 10.3389/fped.2026.1779054 (PMC12999782; doi:10.3389/fped.2026.1779054)

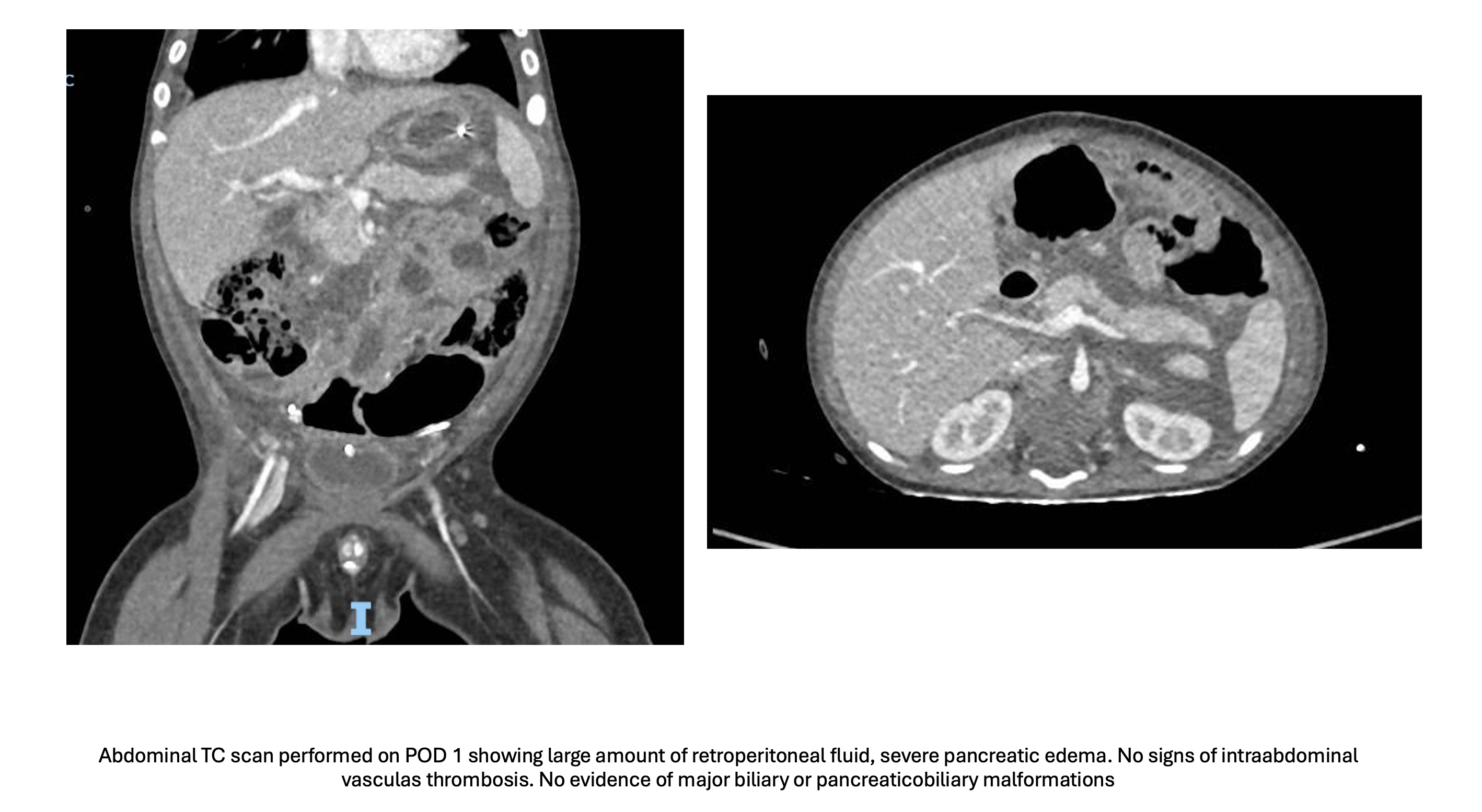

Supplement: Supplementary file 2 [file Image1.tiff]
